# Comparative studies of critical physiological limits and vulnerability to environmental extremes in small ectotherms: How much environmental control is needed?

**DOI:** 10.1111/1749-4877.12297

**Published:** 2018-07-26

**Authors:** Ary A. HOFFMANN, Carla M. SGRÒ

**Affiliations:** ^1^ School of BioSciences, Bio21 Institute The University of Melbourne Melbourne Australia; ^2^ School of Biological Sciences Monash University Melbourne Australia

**Keywords:** comparative studies, common garden, critical limits, ectotherms, environmental control, thermal limits, stress resistance

## Abstract

Researchers and practitioners are increasingly using comparative assessments of critical thermal and physiological limits to assess the relative vulnerability of ectothermic species to extreme thermal and aridity conditions occurring under climate change. In most assessments of vulnerability, critical limits are compared across taxa exposed to different environmental and developmental conditions. However, many aspects of vulnerability should ideally be compared when species are exposed to the same environmental conditions, allowing a partitioning of sources of variation such as used in quantitative genetics. This is particularly important when assessing the importance of different types of plasticity to critical limits, using phylogenetic analyses to test for evolutionary constraints, isolating genetic variants that contribute to limits, characterizing evolutionary interactions among traits limiting adaptive responses, and when assessing the role of cross generation effects. However, vulnerability assessments based on critical thermal/physiological limits also need to take place within a context that is relevant to field conditions, which is not easily provided under controlled environmental conditions where behavior, microhabitat, stress exposure rates and other factors will differ from field conditions. There are ways of reconciling these requirements, such as by taking organisms from controlled environments and then testing their performance under field conditions (or vice versa). While comparisons under controlled environments are challenging for many taxa, assessments of critical thermal limits and vulnerability will always be incomplete unless environmental effects within and across generations are considered, and where the ecological relevance of assays measuring critical limits can be established.

## INTRODUCTION

Climate change is expected to result in a rapid increase in the frequency of extreme climatic events (IPCC 2016). As a consequence, there is increasing interest in measuring the resistance of small ectotherms to climatic extremes, because such periods have substantial effects on the distribution and abundance of these organisms (Hoffmann & Parsons 1991; Overgaard *et al.* 2014; Garcia-Robledo *et al.* 2016). While the impact of climate change on small ectotherms is likely to be complex, involving a range of factors including biotic interactions exerting indirect effects (van Asch *et al.* 2013; Wong & Daniels 2017), the direct effects of extremes on organisms is likely to be an important risk factor in local extinction of populations (Sunday *et al.* 2011; Overgaard *et al.* 2014).

A number of small (Calosi *et al.* 2010; Mitchell & Hoffmann 2010; Sgrò *et al.* 2010; Nyamukondiwa *et al.* 2011) and larger (Kellermann *et al.* 2012b; Kaspari *et al.* 2015; Garcia-Robledo *et al.* 2016) scale comparisons of responses of small ectotherms to extremes have been undertaken across multiple populations and organisms to evaluate the relative susceptibility of taxa to current and future climates. These studies include comparisons of tropical versus temperate species (Kellermann *et al.* 2012a) and populations (Sgrò *et al.* 2010; van Heerwaarden *et al.* 2016a), invasive versus endemic species (Janion *et al.* 2009), and low versus high elevation species and populations (Garcia-Robledo *et al.* 2016) as well as comparisons of species sampled from different habitats at the same geographic location, such as exposed and shaded surfaces (Kaspari *et al.* 2015). These comparisons typically focus on the upper and lower temperatures resulting in mortality (upper or lower lethal limits, ULT, LLT) or resulting in the loss of mobility or coordination of animals (critical thermal limits, CTmax or CTmin) and are sometimes also measured using respiration (de la Vega *et al.* 2015). Other limits that have been considered in small ectotherms include upper and lower growth limits (Deutsch *et al.* 2008), limits associated with dry conditions (Kellermann *et al.* 2009) and limits associated with chronic rather than acute exposures defined in terms of their impact on fitness traits such as mating and development (Rako & Hoffmann 2006; Magozzi & Calosi 2015). Thermal and aridity limits are usually measured by monotonically increasing levels of stressful conditions. However, they have also been measured through responses to altered levels of thermal variability investigated in both natural (Kristensen *et al.* 2008) and laboratory environments (Zhao *et al.* 2014).

Thermal limits of small ectotherms depend on the nature of the response trait being measured, the exposure period and the rate at which stress is applied (Hoffmann 2010). Limits based on reproduction are typically much narrower than those based on lethality (Piyaphongkul *et al.* 2012) and increasing exposure times tend to decrease them. Limits defined by sublethal effects can depend on exposures across as well as within generations (Guo *et al.* 2013) and on the strength of the biotic interactions to which an organism is exposed, which can result in the displacement of one species by another even when temperatures are well below lethal limits (Davis *et al.* 1998). Limits vary markedly as a result of the way organisms are treated prior to being exposed to thermal extremes, as well as the way in which stresses are imposed (Terblanche *et al.* 2011). Given all these sources of variability, critical upper or lower temperatures for an organism need to be defined with reference to the length and rate of exposure to thermal stress, nature of the fitness effect being measured, past history of an organism, and biotic context.

Researchers tend to use standardized and repeatable assays of resistance when assessing vulnerability (Moretti *et al.* 2017), particularly for CTmax, CTmin or mortality measures. However, these measures might not be the most ecologically relevant. Even when standardized assays are used, the degree of environmental control to which taxa are exposed has varied markedly between studies (see Table 1). At one extreme, this involves tightly controlling culture conditions of populations or species over one or more generations, as well as comparing taxa under highly controlled environments (Kellermann *et al.* 2012a). On the other hand, comparative studies of taxa also include cases where individuals are directly collected from the field (Bishop *et al.* 2017; Hemmings & Andrew 2017). Control can also fall between these approaches, such as experiments where field-collected organisms are held for a period under controlled conditions to acclimate them prior to testing (Chown *et al.* 2007; Calosi *et al.* 2010).

The results of tests both with and without environmental control have been interpreted as measuring the susceptibility of organisms to climate extremes, and used to predict vulnerability for a whole range of traits and scenarios. For instance, in assessing the impact of thermal limits on the distribution of bugs spreading Chagas disease, de la Vega *et al.* (2015) focused on laboratory colonies reared for multiple generations under controlled conditions; in contrast, Verhoef *et al.* (2014), investigating the effects of thermal extremes on the distribution of biting midges, another group of disease vectors, considered midges that were collected directly from the field.

Which of these approaches is the right one to use?

**Table 1** Some examples of invertebrate studies where different levels of environmental control are used when undertaking population and species comparisons

| Organism and context | Level of environmental control | Traits compared | Reference |
|---|---|---|---|
| 1 Ant species across elevation gradient | None, differences due to the environment and inherent variation | Body temperature and CTmin, CTmax | Bishop *et al.* 2017; Hemmings & Andrew 2017 |
| 2 Rolled-leaf beetles across tropical elevation gradients | None, as in 1. | CTmax | Garcia-Robledo *et al.* 2016 |
| 3 Invasive and non-invasive springtail species | Intermediate, springtails held for 7–10 days. Early developmental or cross generation effects not controlled. | Desiccation resistance following temperature acclimation. | Chown *et al.* 2007 |
| 4 European diving beetles from the genus *Deronectes* | Intermediate, beetles held for 7 days, as in 3. | UTL, LTL | Calosi *et al.* 2010 |
| 5 *Drosophila* species from across the globe | High, *Drosophila* species cultured under the same conditions for multiple generations | Adult resistance to desiccation, CTmin, CTmax | Kellermann *et al.* 2012a, 2012b |

CTmax, upper critical thermal limit; CTmin, lower critical thermal limits; LLT, lower thermal limit; ULT, upper lethal limit.

And how does this relate to all the other sources of variability in testing endpoints and contexts mentioned above? Below we emphasize that the correct approach is likely to depend on the hypothesis being tested, which needs to be clearly stated. Our purpose in this paper is, first, to spell out some limitations and benefits of the approaches currently applied to thermal limits and, second, to identify opportunities for future work. In considering these issues, we emphasize the standard quantitative genetic model for analyzing phenotypic variation, which was considered in a recent review of thermal plasticity in insects (Sgrò *et al.* 2016) and helps to focus on hypotheses and assumptions. The phenotype of an organism (P) (such as an organism's CTmax) defined within all the complexities mentioned above is considered a summation of inherent genetic (G) and environmental (E) effects with variation resulting from these effects being defined as $V_G$ and $V_E$, respectively, both of which are also subject to maternal effects due to the environment ($M_e$) and inherited maternal factors passed on through the maternal lineage, such as mitochondria and *Wolbachia* ($M_g$). These cross-generation environmental effects might persist for several generations if epigenetic mechanisms are responsible. E includes a complex of short-term hardening, longer-term acclimation and developmental rearing effects on thermal resistance and may be negative or positive, depending on whether they increase or decrease fitness (i.e. the beneficial acclimation hypothesis [Huey *et al.* 1999] or cost of acclimation hypothesis [Hoffmann 1995]). Finally, genetic variation for plasticity (most often reported as genotype-by-environment interactions, or GxE) may also exist for the traits in question, and must also be considered when assessing the importance of E on P (Sgrò *et al.* 2016).

## ASSESSING LIMITS WITH WEAK ENVIRONMENTAL CONTROL

Recent large-scale studies with ants highlight cases where environmental control of taxa being compared is minimal. Ants are collected from an environment or tested *in situ*, exposed immediately to a stressful condition in a water bath or dry apparatus and then typically tested for their critical thermal responses at the upper and lower end by scoring loss of mobility or ability to maintain some other function (Andrew *et al.* 2013; Garcia-Robledo *et al.* 2016; Bishop *et al.* 2017). In these comparisons, the assays themselves can be tightly con-

**Table 2** Hypotheses around vulnerability that can be tested with different approaches (and where caution is required)

| Hypothesis being tested | Level of culture control required | Level of test control required | Potential inappropriate context | Example |
|---|---|---|---|---|
| 1 Species occur close to safety margins as a consequence of G and E. | Minimal, although it is important to capture seasonal variation and issues related to microhabitat. | High, and measures need to be linked to natural environment exposure levels | Comparisons where there is tight environmental control not relevant to field context | Sunday *et al.* 2014; Boardman & Terblanche 2015 |
| 2 Plastic changes influence fitness under environmental extremes | Moderate to high, to ensure that plastic effects are accurately defined. | Low, changes in fitness might be scored indirectly such as in field releases | Costs and benefits of plastic changes can be difficult to evaluate because they are context dependent. | Kristensen *et al.* 2008 |
| 3 Plastic changes at a particular life stage affect stress resistance, including across generations | Moderate to high, ideally the effect of a particular mechanism is tested within the context of other sources of environmental and inherent variability | High | Contexts where environmental control is too tight may lead to an overestimate of importance of a particular effect. | Zhang *et al.* 2015; Kellermann *et al.* 2017 |
| 4 There is an interaction between basal resistance and plasticity | High | Medium to high | Although this issue is often tested with only partial environmental control, this can lead to incorrect characterization of basal resistance | Nyamukondiwa *et al.* 2011 |
| 5 There is phylogenetic signature in responses to extremes | High. Organisms should be cultured across the same controlled conditions, preferably involving 2 or more generations prior to comparison, to ensure that differences do not simply reflect environmental effects | High to ensure repeatable results | Direct comparisons of CTmax, CTmin from the field, where there is no or little control. | Kellermann *et al.* 2012; Garcia-Robledo *et al.* 2016; Comte & Olden 2017 |
| 6 Particular genes, genetic constructs, metabolic and transcriptomic changes underlie variation in vulnerability | High, so that E can be controlled and G isolated. However, it may also be desirable to eventually test genetic constructs in the presence of multiple sources of variation within a population | High | For transcriptomic and metabolic studies in particular, any study that does not rigorously control the environment or state of the organism (including age and developmental stage) given that these profiles change markedly for a variety of causes. | Fabian *et al.* 2012; Chung *et al.* 2014; Griffin *et al.* 2017 |
| 7 Genetic variability controls the ability of organisms to evolve and alter levels of vulnerability | High, to ensure that G can be separated from E, unless genetic relatedness within populations can be used to estimate genetic components | High, to ensure that traits are consistently assessed | Few studies have considered comparative analysis of heritable variation across species, most are single species comparisons. | Kellermann *et al.* 2009 |
| 8 Trait interactions lead to costs and tradeoffs associated with vulnerability | High, otherwise effects might be due to differences in environmental exposure rather than tradeoffs per se | High, otherwise partitioning not possible | Trait interactions depend on context which might not be captured. | Bujan *et al.* 2016 |

trolled and tested for repeatability, but no or little attempt is made to standardize the organism's environment prior to testing. These assessments can be readily carried out and include any effects of E (acclimation, hardening and cross-generation effects) whether beneficial or detrimental as well as inherent differences in resistance due to G. Typically, specimens are collected from the field without regard to the microenvironment where they are found, although there are exceptions (Feder *et al.* 1997).

This approach can be powerful for testing hypotheses on the extent to which P of different species might be approaching the thermal safety margins (Table 2, Hypothesis 1): the CTmin/CTmax (or UTL, LTL) values provide a picture of the proximity of the inherent resistance of the species tested to limits they might experience. It has, therefore, been used to assess whether margins are higher or lower in tropical or temperate environments (Sunday *et al.* 2012) or whether critical margins are exceeded such that behavioral avoidance of stressful conditions is implied (Sunday *et al.* 2014).

Such conclusions, however, assume that the thermal environment of a taxon can be accurately defined within a location across multiple life stages. It may be possible to test these assumptions in larger ectotherms such as lizards where animals can be tracked and body temperature measured regularly to characterize their thermal environment (Huey *et al.* 2009). However, for small invertebrates, there is typically a level of uncertainty around many aspects of their life cycles. For instance, the natural breeding sites of many *Drosophila* species, apart from cosmopolitan species that are human commensalists, is poorly defined with only a few exceptions (Jenkins & Hoffmann 2001; Parkash *et al.* 2013), such that their thermal environments are often unknown.

Defining the environment experienced by organisms is critical because differences in E experienced by taxa within the same location can be substantial. For instance, variation in CTmax in ants depends on the microhabitat used by a species, being relatively higher in ant species that are most active above ground (Baudier *et al.* 2015). Surface dwelling ants exposed to shade have CTmax values that are 3.5–5 °C lower than those of canopy ants (Kaspari *et al.* 2015), while resistance to desiccation is also affected by these habitat differences (Bujan *et al.* 2016). Microhabitat features that are used differently by species can further modify temperature responses, such as the extent to which ants are exposed to sun flecks through the forest canopy (Spicer *et al.* 2017) and spiders are exposed to shading from rocks (van den Berg *et al.* 2015). Stress exposure is also influenced by diel patterns of activity of a species (Verble-Pearson *et al.* 2015).

Finally, when characterizing the resistance of population or species from a site, it is often assumed that the thermal or desiccation responses of the individuals being characterized are representative of that taxon. In practice, conspecifics sampled from nearby locations can differ markedly in thermal responses, as evident in grasshoppers (Slatyer *et al.* 2016). Such variation may reflect a range of factors, including microhabitat variation, genetic differences, or even carry over effects across generations. If taxa are not resident at a location, then thermal responses might be influenced by the environment experienced by the maternal generation (i.e. $M_e$). The extent to which focal individuals are resident at the location of capture/assessment needs to be verified such as through mark release recapture studies and/or the use of molecular markers to measure relative rates of movement across generations.

While a lack of environmental control may be appropriate for comparing thermal limits of species as long as the above assumptions are met, this approach will not necessarily indicate inherent resistance levels of different species (i.e. genetically based differences, G), which are needed when testing hypotheses around phylogenetic signal (Table 2, Hypothesis 5) or for linking differences in gene expression, metabolism and other mechanisms to differences in thermal resistance among species (Table 2, Hypothesis 6). This is simply because G cannot be separated from E when comparing species from different locations or from the same location with different microhabitats. Where the impact of phylogeny on traits is, therefore, explored in such comparisons (e.g. Garcia-Robledo *et al.* 2016), any interpretations should be made cautiously because any apparent phylogenetic signature might reflect factors other than evolutionary conservatism of thermal limits. Related organisms might have similar CTmax values not because of phylogenetic constraint, but because they are more likely to live under similar thermal conditions that, in turn, influence CTmax through hardening and developmental acclimation. An apparent phylogenetic signature then emerges as a consequence of E rather than a shared evolutionary history.

## ASSESSING LIMITS WITH PARTIAL ENVIRONMENTAL CONTROL

In order to at least in part control for differences in E

between species or at least be able to measure the magnitude of E, two approaches are commonly used: testing specifically for environmental variability likely to influence thermal responses (Garcia-Robledo *et al.* 2016; Slatyer *et al.* 2016), or undertaking experiments where some component of E is also measured (Chown *et al.* 2007; Calosi *et al.* 2010). In the first approach, variability might be measured and controlled statistically through assessing the geographical (or environmental) proximity from where taxa were sampled, or by comparing the responses of multiple samples of taxa from different environments. For instance, in a comparison of beetles along an elevation gradient, Garcia-Robledo *et al.* (2016) included widespread and narrowly distributed species in their comparison and showed that the widespread species had high CTmax regardless of where samples originated (high or low elevations), whereas species restricted to high elevations had lower CTmax than those restricted to lower elevations; in this case, there is an indirect argument for differences between the narrowly distributed species representing inherent differences (G) in CTmax rather than environmental effects (E). This assumes, first, that the widespread and more restricted species from the same elevation share any environmental conditions that result in plastic responses and, second, that the widespread species do not have high rates of movement across the elevation gradient. However, variation in upper thermal limits of mountain grasshoppers distributed across an elevation gradient largely partitions within species rather than across species (Slatyer *et al.* 2016); the high level of variation among samples from different elevations, which may reflect local adaptation within a taxon or local plastic responses, needs to be understood when comparing the vulnerability of species.

In the second approach, attempts are made to provide partial environmental control by holding individuals (of a single life stage, often adults) from the focal species or populations under experimental conditions for a period of a few hours or days and then assessing whether these treatments have influenced thermal resistance (Chown *et al.* 2007; Calosi *et al.* 2010; Slatyer *et al.* 2016). This approach can be useful in capturing one aspect of thermal acclimation (usually sublethal exposure to a constant temperature for a period) but is insufficient to capture the full range of plastic effects due to E. One reason is that thermal resistance can represent a culmination of effects across multiple life stages; for instance, adult cold recovery in the vinegar fly (*Drosophila melanogaster* Meigen, 1830) increases markedly as a consequence of a combination of larval developmental acclimation, early adult acclimation, and adult hardening as a consequence of a short exposure period (Colinet & Hoffmann 2012). Similarly, resistance to desiccation stress is likely to increase depending on a combination of plastic responses triggered during development (Parkash & Ranga 2014) as well as at the adult stage (Hoffmann 1990). Stressful conditions at earlier life stages are particularly likely to have negative effects on a range of fitness-related traits (Zhao *et al.* 2014). Negative effects can also be triggered by stressful conditions experienced in the previous generation (Carriere & Boivin 2001; Magiafoglou & Hoffmann 2003). This level of complexity, which is being increasingly investigated (Sgrò *et al.* 2016), is not captured in experiments that only assess plastic responses by acclimating field-caught focal individuals for a few days at a constant temperature prior to assessing thermal responses.

In addition to testing for some aspects of plasticity, experiments that involve partial environmental control have also been used to test hypotheses around the evolution of plasticity, and the extent to which plasticity constrains or facilitates trait evolution. For instance, Calosi *et al.* (2010) acclimated field-caught individuals to assess whether acclimation capacity constrains the basal thermotolerance of diving beetles, and found a positive association between upper thermal tolerance and its acclamatory ability. This suggests that species with the lowest tolerance to high temperature are also at the highest risk due to low plasticity. This situation contrasted with a negative relationship between upper thermal limits and plasticity in prawns which was also established from field-caught material (Magozzi & Calosi 2015), although the fact that Calosi *et al*. (2010) assessed thermal limits using 2 different starting temperatures may in part explain the consistency in results.

Associations between basal resistance and plastic responses may well vary, depending on the type of plastic responses being investigated. Recent work shows that thermal conditions experienced during development can have lasting effects on adult thermotolerance that persist regardless of adult acclimation treatments (Kellermann *et al.* 2017). To fully explore the association between plasticity and basal resistance, environmental conditions need to be controlled and potential effects considered across all life stages (and perhaps across generations to control for maternal effects as well). These points are especially pertinent when meta-analyses are used to assess theories around the evolution of plasticity (Gunderson & Stillman 2015; Comte & Olden 2017) and its role in mediating climate change risk (Gunderson *et al.* 2017). Much of the data used in such meta-analyses comes from diverse experimental approaches, and many studies with very little or no environmental control, and/or with environmental effects considered across only a limited set of developmental stages. While the use of specific terms such as hardening or developmental acclimation might help reduce the uncertainty when comparing results across studies, even these terms can be ascribed different meaning by different researchers. For example, developmental acclimation is often used to denote any irreversible plastic change, rather than a plastic shift in response to early-life conditions or plasticity in a particular life stage. To minimize such confusion, "plasticity" should always be defined in terms of the life stages examined, and whether the plastic change is reversible or not.

## ASSESSMENTS WITH STRONG ENVIRONMENTAL CONTROL

In these comparisons, the impact of specific genetic variants on resistance to extremes is often assessed with the effects of E minimized so that the effects of G become more evident. The aim here is to minimize the likelihood that environmental noise will obscure the impact of genetic variants on focal traits. However, the downside is that the relative importance of G might be overestimated in the absence of environmental effects. For instance, a high level of environmental control is often used to quantify the impact of specific alleles in isogenic backgrounds, such as in the case of a fatty acid synthase gene on desiccation resistance in the rainforest fly *Drosophila birchii* Dobzhansky & Mather, 1961 (Chung *et al.* 2014), or to investigate the genomic basis of quantitative genetic variation, such as desiccation resistance in *D. melanogaster* (Griffin *et al.* 2017). Yet it is then not clear if these variants have much impact on traits in heterogeneous environmental backgrounds, where trait heritabilities are typically lower. Environmental variation can have a particularly large impact on G when there are strong GE interactions that change genotype rankings across environments (Kobey & Montooth 2013).

Environmental control is needed to separate different sources of environmental variation and their relative importance (Table 2, Hypothesis 3). Tight control is required when assessing whether the impact of stress at one life stage might influence later stages (Zhang *et al.* 2015). Breeding experiments across multiple generations are needed to identify negative as well as positive cross generation effects on thermal responses (Magiafoglou & Hoffmann 2003; Sgrò *et al.* 2016). Typically, in cross generation experiments, organisms are reared under more than one set of constant conditions and offspring are then tested under the same conditions in a design where all alternatives are considered (Marshall & Uller 2007). Within a generation, tight environmental control is required when separating different sources of plasticity, such as when comparing rearing acclimation, developmental acclimation and hardening effects as undertaken in *Drosophila* populations (Kellermann *et al.* 2017). However, when the effects of plasticity are evaluated on vulnerability within a field context (Table 2, Hypothesis 2), control of test conditions can be relaxed.

Environmental control is important if levels of genetic variation are being characterized (Table 2, Hypothesis 7). Populations can evolve rapidly in response to climate change, including evolutionary changes in resistance to thermal extremes (e.g. Geerts *et al.* 2015). The impact of these evolutionary shifts depends on heritable levels of variation within populations and species as well as the extent to which traits are genetically correlated with each other (Hoffmann & Sgrò 2011). With sufficient knowledge of heritable variation, predictions can be made about the extent to which communities can persist under climate change (Bush *et al.* 2016). Evolutionary shifts have major implications for all aspects of vulnerability prediction because they effectively mean that vulnerability is not a constant property of species but is a characteristic that is likely to vary through time, as environments change and as organisms adapt. Consequently, attempts are being made to empirically quantify the adaptive capacity of organisms across different environmental contexts; that is, to separate the effects of E and G on critical thermal limits under environmental conditions that reflect projected changes in climate. Some studies have been performed in the wild, with no environmental control, but have instead used the Animal Model approach to partition G from E in wild animal populations (e.g. in birds, Husby *et al.* 2011), others have used the offspring of field-caught individuals under controlled laboratory conditions to assess adaptive potential (e.g. in tube worms and reef fish, Chirgwin *et al.* 2015; Munday *et al.* 2017), while many others have been undertaken with populations that have been reared for multiple generations in the laboratory, allowing for a high level of environmental control when partitioning E from G require (van Heerwaarden *et al.* 2016b). Unfortunately, it is generally not possible to follow small ectotherms as individuals and their offspring within field populations, precluding the use of Animal Models in data from natural populations.

Genetic studies can also be used to explore issues around the nature of interactions influencing the evolution of traits (Table 2, Hypotheses 4 and 8), such as costs of thermal and desiccation resistance and the trait-specific nature of genetic effects and the extent to which different measures of thermal responses are genetically independent (Gerken *et al.* 2016). These patterns, in turn, can inform comparative studies about evolutionary constraints acting on specific traits (Kellermann *et al.* 2012a).

## CHALLENGES IN DEFINING APPROPRIATE ENVIRONMENTS (AND POPULATIONS)

Although comparative studies can be carried out under controlled environmental conditions by rearing species or populations in the same environments, there is no guarantee that the same environment will be equally favorable to different species or populations. One taxon may be pre-adapted to laboratory conditions, and, thus, be under a low level of stress and easy to rear. A different taxon being used for a comparison might be difficult to rear under the same conditions, and be highly stressed. For instance, tropical *Drosophila* species and populations often perform more poorly under cooler conditions than temperate populations and species (David *et al.* 2005; Trotta *et al.* 2006). Differences in suitability could, in turn, influence thermal responses and CTmax or CTmin if some taxa are stressed by rearing conditions and others are not. The relative resistance of species or populations might then shift depending on the conditions being used to impose environmental control. This problem is not easily circumvented but ideally should be checked by rearing the species or populations being compared under multiple controlled conditions (Hoffmann *et al.* 2005). The issue is further compounded by the presence of microbiota within species that are affected by rearing environments and might influence stress resistance, although this is an area that needs further investigation (Sgrò *et al.* 2016).

Environmental control also raises issues around the choice of stock population used for characterizing responses and the level of replication of stocks required to generate confidence in results. Where model small ectotherms are used, it is often easy to source material from stock centers (such as in *Drosophila* [Kellermann *et al.* 2012b; Nyamukondiwa *et al.* 2011]) or to use other well-established laboratory stocks. However, the stocks might differ in levels of inbreeding and the extent of laboratory adaptation, both of which may influence stress resistance (Hoffmann *et al.* 2001; Griffiths *et al.* 2005). This issue is likely to be particularly important for species that are difficult to rear under artificial conditions. Such species will typically need to undergo an intense period of laboratory adaptation and subsequent bottlenecks (Stuart & Gaugler 1996), which may make interpretation of experimental results difficult when trait means are altered as a consequence of adaptation.

A third issue is that environmental control might require multiple "controlled" conditions to be considered if there are strong interactions between taxon vulnerability and the environment. A taxon with relatively high vulnerability under one set of conditions might be relatively resistant under a different set of conditions. Such interaction effects have been highlighted in species comparisons (Davis *et al.* 1998) and emphasize the importance of characterizing differences across a range of environments linked to those that the taxa experience within the context of their natural environment.

## RELEVANT VULNERABILITY ENDPOINTS

Numerous measures of resistance have been developed and used to assess thermal limits in small ectotherms (Hoffmann *et al.* 2003; Castaneda *et al.* 2015; Moretti *et al.* 2017). Regardless of the extent of experimental control, a challenge for all studies assessing thermal limits and their plasticity is the choice of endpoint used, and their relevance to the ecological context of the organism being studied. This means considering the relevance of all aspects of environmental control discussed above with respect to natural situations, including exposure times and speeds at which thermal conditions are experimentally changed, the conditions that are experienced prior to assessment within and across generations, and the state of the organism (Colinet *et al.* 2015). However, it also means considering the broader fitness consequences of the measures being taken; if non-lethal temperatures induce non-reversible sterility (David *et al.* 2005), this measure may be more relevant to the ecology of an organism than UTL.

The many endpoints used to measure thermal resistance include mortality, knockdown from standing positions, loss of muscle coordination or activity, recovery after exposure to acute stress and fitness components after recovery (reviewed in Hoffmann *et al.* 2003; Castaneda *et al.* 2015). Recovery from cold is generally reversible, although there is often mortality and reduced reproductive output after exposure (Jenkins & Hoffmann 1999). Inactivation from heat usually results in eventual death of small ectotherms. If mortality is not the desired endpoint, heat resistance must be scored in different ways, such as by using long knockdown tubes that generate a thermal gradient and from which flies can be recovered as they fall through the tube (Gilchrist & Huey 1999). Other criteria can also be used, depending on the species. In ants, it is possible to monitor behavioral responses to hot areas and to determine at what temperature these areas are abandoned (Spicer *et al.* 2017). It is also possible to measure body temperature directly in live and dead individuals to see if thermal limits are exceeded (Hemmings & Andrew 2017). Instead of measuring CTmax visually, it may also be possible to measure it through changes in metabolism as in bed bugs (de la Vega *et al.* 2015), which can be a useful approach for species of a small size. Regardless of the method used, however, the assessments are assumed to reflect how thermal stress impacts fitness in nature, and not all studies have directly demonstrated such a link.

Assessments of thermal limits typically involve placing individuals in a heating or cooling environment of some sort. This might be a water or ethylene glycol/water mix bath or a dry bath where temperature changes are evened out through the use of an aluminium heating block (e.g. Bishop *et al.* 2017). Other more novel approaches are also being used such as PCR machines where temperature cycles can be very accurately manipulated and complex cycles used (Kong *et al.* 2016).

In addition to decisions around which endpoints should be used, the method used and type of exposure will also impact estimates of thermal limits. Thermal tolerance can be assessed using either static (constant) assays (Hoffmann *et al.* 2003) or dynamic (ramping) assays that involve gradually heating or cooling an animal from a particular starting temperature until the specific endpoint is reached (Terblanche *et al.* 2011). Ramping assays are argued to be more ecologically relevant because they are thought to better reflect changes in temperature in the field, and because they indicate the activity range for a population under acute conditions experienced in nature. However, the rate of change in temperature used in these assays will influence estimates of CTmax and CTmin values quite markedly, as shown in flies (Sgrò *et al.* 2010), ants (Bentley *et al.* 2016) and other insects (Terblanche *et al.* 2007). Based on exposure temperature changes in the field (Terblanche *et al.* 2011), rates of 1 °C per minute are typically used (Schou *et al.* 2017), although higher ramping rates are also applied; for instance, Bishop *et al.* (2017) applied a consistent ramping rate of 1 °C every 3 min when characterizing CTmin and CTmax of ants along an elevation gradient.

Ideally, rates should aim to reflect those found in nature but this can be hard to simulate or even score accurately when behavior influences the temperatures to which organisms are exposed. For this reason, comparative analyses typically adopt a standard rate of ramping (or direct exposure) in the hope that the same test can at least reflect the relative resistance of different species or populations. However, the ranking of populations and species with respect to CTmax/CTmin can depend on the nature of the test and ramping rate applied, particularly for CTmax (Mitchell & Hoffmann 2010; Castaneda *et al.* 2015). Slower ramping rates can lead to longer exposure times and potentially greater stress levels (Castaneda *et al.* 2015), as seen in several species (Chidawanyika *et al.* 2017), although slower rates might also increase the opportunity for acclimation that can increase resistance (Overgaard *et al.* 2014), so predictions are not necessarily straightforward.

Beyond effects on means, predictions about the evolutionary capacity of thermal limits can also vary with methodology (Chown *et al.* 2009; Mitchell & Hoffmann 2010; Rezende *et al.* 2011). Slow ramping rates can result in low heritability and additive genetic variance for heat tolerance (Mitchell & Hoffmann 2010; Blackburn *et al.* 2014) (although this is not always the case [van Heerwaarden & Sgrò 2013]), which implies that evolutionary responses to selection imposed by gradual heating might be constrained. However, adaptive responses to selection will depend not only on genetic variation in single traits but also on the covariation between multiple traits under selection (Kelly *et al.* 2013, 2016; Blackburn *et al.* 2014). Thus, using one endpoint to assess heat tolerance may underestimate or overestimate adaptive potential if different measures of thermal limits share a genetic basis. For example, while van Heerwaarden and Sgrò (2013) found that slow ramping and static measures of heat tolerance have significant levels of genetic variation and are genetically correlated in *D. simulans*, Blackburn *et al.* (2014) found that they were not genetically linked in *D. melanogaster*, and that adaptive responses to gradual increases in temperature were constrained by very low levels of genetic variation. However, when artificial selection was applied to select directly on static heat tolerance, and correlated responses in ramping heat tolerance were then assessed, Hangartner and Hoffmann (2016) found that all components of heat tolerance responded to selection, meaning that they were at least partially genetically correlated. This suggests that a general mechanism partly underpins static measures of heat resistance, as well as those involving ramping. These results suggest, first, that different measures of heat tolerance may, nonetheless, provide similar insight into the adaptive capacity of populations (in contrast to species comparisons), and, second, that selection experiments, rather than family studies, are perhaps more powerful ways of detecting and assessing adaptive capacity, particularly when traits harbor low but evolutionarily significant levels of genetic variance.

Unlike for CTmax, there is consistency in how *Drosophila* species respond to environmental conditions with respect to CTmin. Shou *et al.* (2017) compared 13 species for CTmin when reared under temperatures in the range 12.5 to 30 °C; for CTmin, reaction norms were linear and CTmin increased with increasing developmental temperature across all species, indicating conservation of the reaction norm. This contrasted with CTmax, which showed different responses across the species when cultured at different temperatures; many species increased in CTmax with culture temperature but some decreased or showed no change in CTmax. Thus, differences in methodology may have a smaller impact on outcomes of comparative studies assessing CTmin, rather than CTmax.

Beyond the question of whether ramping versus static assays should be used and which culture temperatures should be applied when testing thermal limits, assessments can also be affected by single or repeated exposures to acute and/or chronic thermal stress. Repeated exposures to sublethal conditions can increase resistance beyond that seen with single exposures (Kingsolver & Buckley 2015), but there can also be costs associated with nighttime warming as seen in aphids (Zhao *et al.* 2014). In addition, fluctuating thermal conditions may increase thermal tolerance (Sorensen *et al.* 2016), although such beneficial effects may be more consistent for lower, rather than upper, thermal limits (Colinet *et al.* 2015).

Changes in environmental factors other than temperature influence assessments of critical thermal limits. For example, the presence/absence of food can increase thermal resistance as in beetles (Chidawanyika *et al.* 2017) but not *Drosophila* (Terblanche *et al.* 2011; Overgaard *et al.* 2012) and other factors such as humidity might also be important (Rezende *et al.* 2011), although not in all cases (Overgaard *et al.* 2012). Food can also decrease CTmax measures as in bed bugs that are more resistant after being starved for 9 days (45.2 °C) compared to 1 day (44.6 °C), although this decreases again with further starvation (de la Vega *et al.* 2015), reinforcing recent work that suggest a complex relationship between changes in body conditions and estimates of CTmax and CTmin (Mitchell *et al.* 2017).

There is evidence that behavior influences thermal limits; different species may modify their behavior such that they are exposed to changed thermal conditions for different lengths of time and, thus, different stress levels (Huey *et al.* 2012; Sunday *et al.* 2014). While the best evidence for behavior playing a significant role in mediating thermal limits comes from reptiles (Sunday *et al.* 2014), there are also examples from insects. For instance, in the meat ant (*Iridomyrmex purpureus* Smith), body temperatures were lower than temperatures in their microclimate, pointing to a high level of behavioral flexibility (Andrew *et al.* 2013). Many domesticated insects, including *Drosophila*, modify their exposure to extremes by utilizing buildings and other structures (Jakobs *et al.* 2015). Other insects evade stressful conditions by entering periods of quiescence or diapause (Sgrò *et al.* 2016). These issues highlight the challenge of linking standard performance curves to conditions experienced in the field and characterizing safety margins.

Finally, size can influence stress resistance and needs to be considered given that size is influenced by environmental factors such as nutrition and temperature as well as inherent differences among taxa. For instance, species differences in size in ants affect both upper thermal limits (Bentley *et al.* 2016) and desiccation resistance (Bujan *et al.* 2016), with resistance levels in both cases increasing with size. In small ectotherms, size can also influence the microclimate experienced by organisms, and, thus, exposure to thermal stress; for example, large size in Panamanian ants decreases the ability of ant species to stay within boundary layers (Kaspari *et al.* 2015), while larger larvae of *Manduca sexta* experience different microclimates on leaf surfaces that increase their exposure to heating and thermal stress (Woods 2013). Thus, while large size might increase intrinsic levels of resistance, it can also increase the level of exposure of the animals to thermal stress.

## WHERE TO NEXT IN VULNERABILITY ASSESSMENTS?

Given the issues outlined above, where do we go next? Clearly there are benefits of carrying out vulnerability assessments on small ectotherms with some degree of environmental control, but how do we then ensure that these are relevant to field conditions? In addition, how can we become confident that relative vulnerability measured directly from the field can help predict the ability of taxa to counter extreme conditions?

Researchers should be looking for opportunities to combine both approaches when assessing vulnerability. For species that can be cultured under laboratory conditions, it is possible to contrast the relative performance of taxa taken directly from the field with their performance in subsequent laboratory generations. This design can be used to test if field assays can detect inherent differences in resistance and to detect environmental effects that carry over across generations (Fig. 1a). Such an approach was used by Schiffer *et al.* (2013); by measuring the CTmax and CTmin of *Drosophila* species taken directly from the field with those obtained in two ensuing generations of laboratory rearing, they showed that species differences in thermal limits were only weakly correlated between field and controlled conditions, and that inherent species differences could only be characterized accurately by controlling for environmental sources of variation and carry-over effects. Sorensen *et al.* (2015) also used a comparison of field caught and laboratory-reared *Drosophila subobscura* Collin, 1936 to show that environmental effects on thermal limits present under field conditions were not consistent with those observed in the laboratory.

Another approach is to work in the opposite direction and consider the performance of taxa reared under controlled laboratory conditions in the field (Fig. 1b). Field releases can be undertaken to test the impact of particular environmental conditions on resistance. *D. melanogaster* flies released under cold conditions exhibit an increased capture rate when they are acclimated to cold conditions, but at a cost when releases are undertaken under warm conditions (Kristensen *et al.* 2008). Similar results were found in field releases using the codling moth, *Cydia pomonella* Linnaeus, 1758 (Chidawanyika & Terblanche 2011). *Trichogramma carverae* Oatman and Pinto, 1987 parasitoids reared under laboratory conditions and hardened by exposure to a non-lethal heat

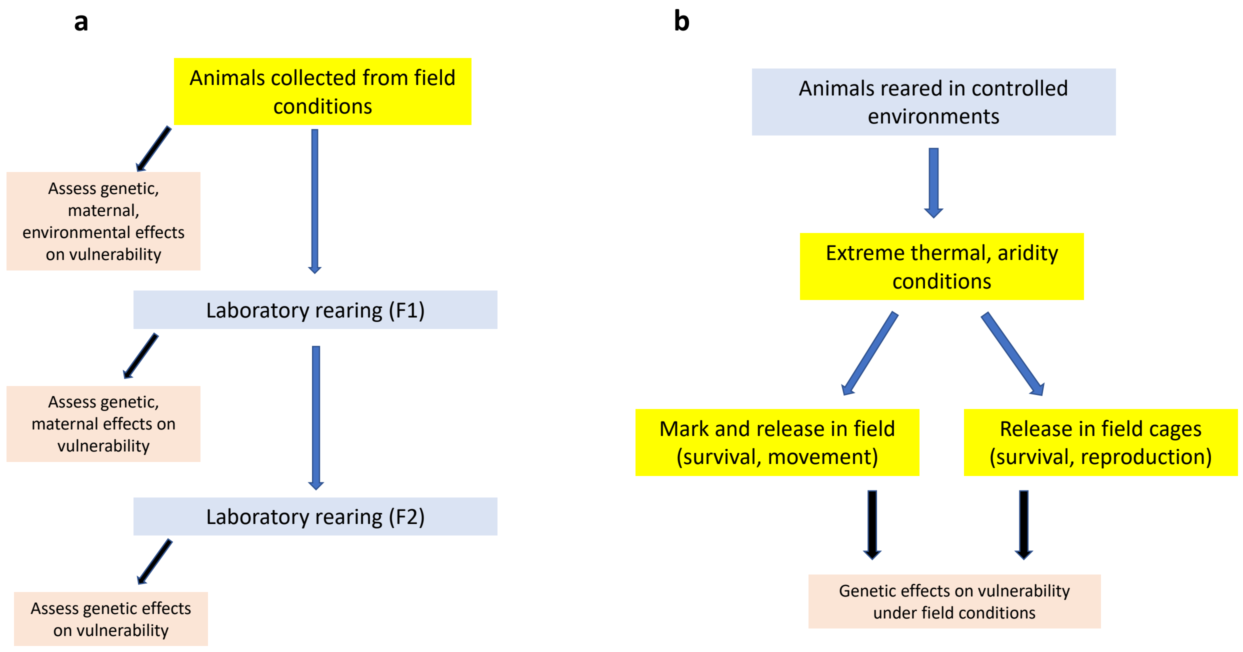

**Figure 1** Assessing vulnerability: Connecting controlled environment studies with fitness evaluations. (a) Cross generation studies of vulnerability that incorporate field and laboratory generations. (b) Field fitness evaluations under extreme conditions using taxa reared under control conditions.

stress show increased parasitism in the field under hot conditions (Thomson *et al.* 2001). Release experiments like these could be used to assess the relative performance of a range of insects; open releases might not be required if insects can be successfully cultured in field cages, an approach that has been used to investigate the response of tropical *Drosophila* to elevation gradients (O'Brien *et al.* 2017) and the ability of spotted wing fruit fly *Drosophila suzukii* (Matsumura, 1931) to persist across winter (Jakobs *et al.* 2015). Such an approach was also used by Nyamukondiwa *et al*. (2013) to assess response of fruit fly species (*Ceratitis capitatsa* and *C. rose*) to thermally varying field conditions.

It will also be important to independently validate vulnerability assessments that indicate differences among taxa. Measures of CTmin or CTmax might be linked to differences in the distribution of species. For instance, Overgaard *et al.* (2014) found that thermal sensitivity (assessed using performance curves) of life history traits in 10 *Drosophila* species was a poor predictor of species distributions, whereas adult thermal resistance assessed using a ramping knockdown assay provided a better predictor. In a larger-scale experiment of almost 100 species cultured under the same controlled environments, Kellermann *et al.* (2012a) linked species differences in CTmin to the climate from which flies were sourced. For CTmax, the signal was weaker but, nevertheless, detectable once humidity was taken into account (Kellermann *et al.* 2012b). Bishop *et al.* (2017) identified variation in CTmin in ants that matched relative abundance of species across an elevation gradient where environmental temperatures varied. In contrast, CTmax in ants has not been clearly linked to species distributions or abundance, although this may reflect the difficulty of characterizing CTmax in ants using loss of mobility as the end-point (Andrew *et al.* 2013).

More studies are needed to consider the extent to which relative rankings of the tolerance of taxa to extremes remains the same when different fitness end-points are applied. For instance, while Andersen *et al.* (2015) showed that 2 out of 5 measures of cold tolerance were better predictors of species latitudinal range, species rankings with respect to cold tolerance were generally similar for 4 out of the 5 measures. These results are consistent with earlier work suggesting that for CTmin it may be that similar rank orders are maintained regardless of the approach used (Kimura 2004). However intra-specific studies show that rankings can vary depending on the methodology used. For instance, across populations of *Drosophila*, patterns of latitudinal variation for upper thermal limits can depend on the assay procedure used (Sgrò *et al.* 2010; Castaneda *et al.* 2015). These types of comparisons need to be extended to incorporate biotic contexts. For instance, vulnerability comparisons under extremes should include competitive interactions among organisms or relative susceptibility to predation.

Should we ignore measures of thermal extremes that cannot be linked to species distributions or the relative abundance of species or populations? Should we regard as less interesting those cases where species have a thermal limit that exceeds conditions they likely experience in the field (e.g. Wu & Wright 2015) or where variation among taxa is particularly small (Mitchell & Hoffmann 2010)? Where there is little variation in resistance among related clades, these might reflect a fundamental physiological limit within an evolutionary clade that restricts a group of species to particular habitats or micro-environments and life cycle within that habitat. Unfortunately, datasets that do not fit *a priori* predictions or that do not demonstrate variability among taxa are likely to be hard to publish and may languish in the drawers of many research laboratories.

The ability to predict the vulnerability of taxa to environmental extremes can be improved by carefully considering the relative importance of environmental and genetic causes of variation in the traits involved. Assessments will always be incomplete unless environmental effects within and across generations can be considered, which will be difficult particularly for field-based research on organisms that cannot be easily reared under controlled conditions. Nonetheless, clearly acknowledging the pros and cons of experimental approaches, and framing assessments of vulnerability within a strong theoretical and hypothesis testing framework will help improve the accuracy of predictions.

## ACKNOWLEDGMENTS

AAH held an NHMRC Fellowship during the completion of this paper.

**Cite this article as:**

## Cite this article as:

Hoffman AA, Sgrò CM (2018). Comparative studies of critical physiological limits and vulnerability to environmental extremes in small ectotherms: How much environmental control is needed? *Integrative Zoology* **13**, 355–71.
